# Activation of peroxisome proliferator activated receptor alpha ameliorates ethanol induced steatohepatitis in mice

**DOI:** 10.1186/1476-511X-10-246

**Published:** 2011-12-30

**Authors:** Lingbo Kong, Weiguang Ren, Wencong Li, Suxian Zhao, Hongmei Mi, Rongqi Wang, Yuguo Zhang, Wenjuan Wu, Yuemin Nan, Jun Yu

**Affiliations:** 1 Department of Traditional and Western Medical Hepatology, Third Hospital of Hebei Medical University, Shijiazhuang, China; 2 Institute of Digestive Disease and Department of Medicine and Therapeutics, Li Ka Shing Institute of Health Sciences, The Chinese University of Hong Kong, Hong Kong

**Keywords:** Peroxisome proliferator activated receptor alpha, ethanol, steatohepatitis, animal experiment

## Abstract

**Background:**

Peroxisome proliferator activated receptor alpha (PPARα) regulates lipids metabolism and inhibits inflammatory response. However, the role of PPARα in alcoholic liver disease is largely unknown. We aim to elucidate the effect and the molecular basis of PPARα in ethanol induced hepatic injury in mice.

**Results:**

C57BL/6J mice fed with 4% ethanol-containing Lieber-DeCarli liquid diet for 12 weeks exhibited hepatocyte steatosis, necrosis and inflammatory infiltration, accompanied with elevated serum alanine aminotransferase (ALT) and aspartic transaminase (AST) levels, decreased hepatic expression of PPARα, lipids oxidation promoting genes and anti-inflammatory factors, as well as enhanced hepatic expression of fatty acids synthesis promoting genes and pro-inflammatory cytokines. Induction of PPARα by PPARα agonist WY14643 treatment for 2 weeks ameliorated the severity of liver injury and restored expression of genes altered by ethanol treatment. However, administration of PPARα antagonist GW6471 for 2 weeks promoted the inflammatory response.

**Conclusions:**

The present study provided the evidence for the protective role of PPARα in ameliorating ethanol induced liver injury through modulation of the genes related to lipid metabolism and inflammatory response.

## Background

Alcoholic liver injury is a progressive process encompassing hepatic steatosis and steatohepatitis. The latter may progress to liver fibrosis, cirrhosis and even hepatocellular carcinoma [1]. Chronic ethanol exposure impairs fatty acid oxidation and enhances lipogenesis by targeting key transcriptional regulators of genes controlling these metabolic processes, including peroxisome proliferators activated receptor gamma coactivator 1 alpha (PGC-1α) [2], sterol regulatory element binding protein 1 (SREBP-1) and its downstream genes, such as fatty acid synthase (FAS) [2], resulting in the accumulation of triglyceride in the liver (steatosis). Fat accumulation renders the liver more susceptible to other injuries. Ethanol also contributes to the up-regulation of pro-inflammatory factors, osteopontin (OPN) [3-5] and cyclooxygenase-2 (COX-2) [6] in the liver, which promotes inflammatory injury and causes alcoholic steatohepatitis. Pharmacological treatment for patient with alcoholic steatohepatitis is still not available. There is compelling need to identify agent to protect liver against ethanol-related inflammatory injury.

Peroxisome proliferator activated receptor alpha (PPARα), interacts with the retinoid X receptor to function as a transcription factor to induce the expression of a series of genes involved in fatty acid transport, mitochondrial fatty acid oxidation, catabolism, and inflammatory responses [7-11]. Down-regulation and/or dysfunction of PPARα are involved in the development of ethanol induced liver injury [11]. However, the role of PPARα in pathogenesis of alcoholic liver disease (ALD) remains largely unknown. In this study, we investigated the effects of PPARα activation in evolution of alcoholic steatohepatitis and the molecular basis of its action in animal experiments.

## Results

### Activation of PPARα by WY14643 lowered the serum levels of alanine aminotransferase (ALT) and aspartic transaminase (AST) in mice fed with ethanol liquid diet

**Figure 1:**
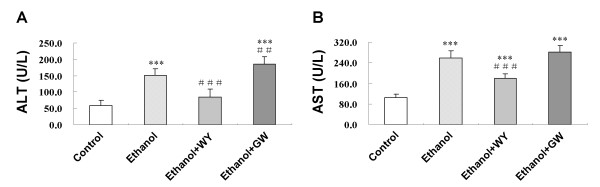
**Effects of ethanol with treatment of WY14643 or GW6471 on serum (A) ALT level and (B) AST level**. Data are expressed as the mean ± SD (n = 6 per group). ***P < 0.001 compared with Control group; ^##^*P *< 0.01, ^###^*P *< 0.001 compared with Ethanol group.

As shown in Figure 1, mice fed with 4% ethanol-containing Lieber-DeCarli liquid diet for 12 weeks showed significantly higher serum ALT and AST levels (*P *< 0.001) compared with Control group, indicating hepatic injury. A significant reduction of serum ALT and AST levels (*P *< 0.001) was noticed after WY14643 treatment. However, GW6471 treatment further elevated the ALT level (*P *< 0.01) than those fed ethanol liquid diet only (Figure 1).

### Activation of PPARα ameliorated liver injury in mice fed with ethanol liquid diet

**Figure 2:**
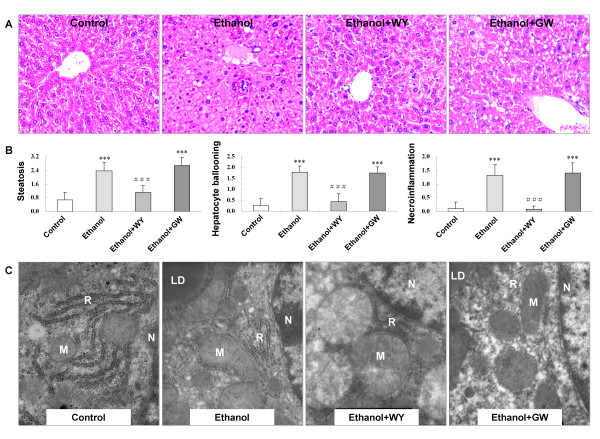
**Changes of liver histopathology and hepatocyte ultrastructure in mice under various treatment conditions**. **(A) **Hematoxylin and eosin stained liver sections from mice liver (Original magnification, ×200), **(B) **Effect of PPARα on scores for hepatic steatosis, hepatocyte ballooning, necroinflammation in ethanol induced liver injury, and **(C) **Electron microscopy for hepatocyte ultrastructure (×20 000). N, nucleus; LD, lipid droplets; M, mitochondria; R, rough endoplasmic reticulum. ***P < 0.001 compared with Control group; ^###^*P *< 0.001 compared with Ethanol group.

The liver sections from mice fed with ethanol-containing liquid diet exhibited disordered lobule structure, hepatocyte ballooning, moderate steatosis, inflammatory infiltration and mild hepatocyte necrosis. WY14643 significantly ameliorated hepatic steatosis and inflammation (*P *< 0.001) (Figure 2).

### Activation of PPARα improved ultrastructural damage of hepatocytes in mice fed with ethanol liquid diet

Rich organelles including mitochondria, endoplasmic reticulum and ribosomes are observed in hepatocytes of normal control livers under electron microscopy. Whilst, in the liver sections of ethanol feeding mice, mitochondrial damage in hepatocytes with broken cristae, ruptured membranes and merged cristae/membranes are observed. Granule fusion and degranulation phenomenons are also found in rough endoplasmic reticulum. These ultrastructural changes in hepatocytes are clearly improved by WY14643 administration (Figure 2C).

### Hepatic expression of PPARα and PPARα-responsive genes in mice fed with ethanol liquid diet and treated with WY14643 or GW6471

**Figure 3:**
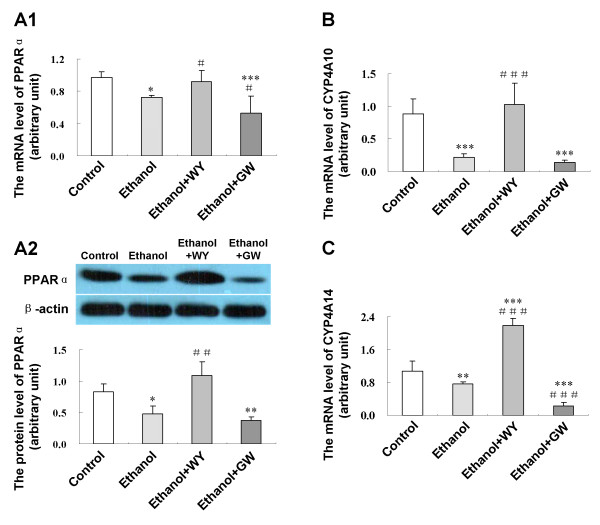
**Effect of WY14643 on hepatic PPARα and PPARα-responsive genes expression in ethanol induced liver injury**. **(A1) **Expression level of PPARα mRNA and **(A2) **protein; **(B) **Expression level of CYP4A10 mRNA; **(C) **Expression level of CYP4A14 mRNA in various treatment groups. Data are expressed as the mean ± SD (n = 6 per group). *P < 0.05, **P < 0.01, ***P < 0.001 compared with Control group; ^#^*P *< 0.05, ^##^*P *< 0.01, ^###^*P *< 0.001 compared with Ethanol group.

As shown in Figure 3, hepatic expression of PPARα mRNA (*P *< 0.05) and protein (*P *< 0.05), as well as PPARα-responsive genes cytochrome P450 4A10 (CYP4A10) (*P *< 0.001) and CYP4A14 (*P *< 0.01) mRNA was down-regulated by ethanol. WY14643 administration increased the expression levels of PPARα (*P *< 0.05), CYP4A10 (*P *< 0.001) and CYP4A14 (*P *< 0.001). However, GW6471 reduced PPARα (*P *< 0.05) and CYP4A14 (*P *< 0.001) mRNA expression.

### Induction of PPARα regulates hepatic expression of lipid metabolism related genes

**Figure 4:**
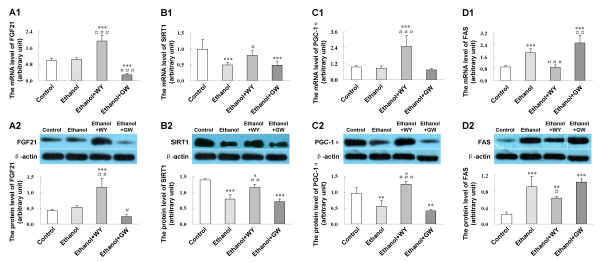
**Effect of PPARα induction by WY14643 on hepatic lipid metabolism related genes expression in ethanol induced liver injury**. **(A1) **Expression level of FGF21 mRNA and **(A2) **protein; **(B1) **Expression level of SIRT1 mRNA and **(B2) **protein; **(C1) **Expression level of PGC-1α mRNA and **(C2) **protein; **(D1) **Expression level of FAS mRNA and **(D2) **protein in various treatment groups. Data are expressed as the mean ± SD (n = 6 per group). *P < 0.05, **P < 0.01, ***P < 0.001 compared with Control group; ^#^*P *< 0.05, ^##^*P *< 0.01, ^###^*P *< 0.001 compared with Ethanol group.

To seek an explanation for the ameliorated hepatic steatosis under WY14643 administration, we assessed the hepatic expression levels of genes involved in lipid metabolism. Relative to control mice, hepatic FAS mRNA (*P *< 0.001) and protein (*P *< 0.001) were enhanced, while sirtuin 1 (SIRT1) mRNA (*P *< 0.001) and protein (*P *< 0.001) as well as PGC-1α protein (*P *< 0.01) were reduced in mice fed with ethanol liquid diet. Administration of WY14643 increased mRNA and protein expressions of fibroblast growth factor 21 (FGF21) (*P *< 0.001 and *P *< 0.01, respectively), SIRT1 (*P *< 0.05 and *P *< 0.01), PGC-1α (*P *< 0.001 and *P *< 0.001), down-regulated FAS expression (*P *< 0.001 and *P *< 0.05) as compared with mice fed ethanol liquid diet only (Figure 4). GW6471 decreased the FGF21 mRNA (*P *< 0.001) and protein (*P *< 0.05) expression, while increased FAS mRNA (*P *< 0.01) expression in mice fed with ethanol.

### Activation of PPARα suppressed hepatic expression of pro-inflammatory factors

**Figure 5:**
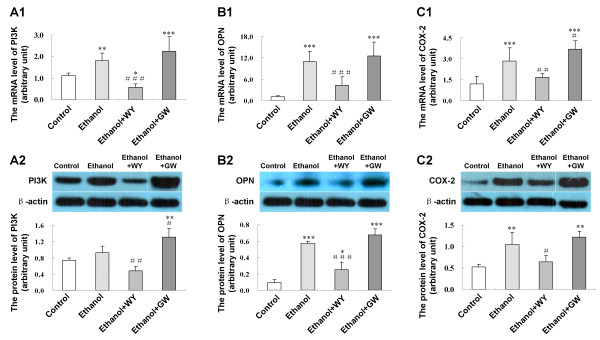
**Effect of PPARα induction by WY14643 on hepatic expression of pro-inflammatory factors**. **(A1) **Expression level of PI3K mRNA and **(A2) **protein; **(B1) **Expression level of OPN mRNA and **(B2) **protein; **(C1) **Expression level of COX-2 mRNA and **(C2) **protein in various treatment groups. Data are expressed as the mean ± SD (n = 6 per group). *P < 0.05, **P < 0.01, ***P < 0.001 compared with Control group; ^#^*P *< 0.05, ^##^*P *< 0.01, ^###^*P *< 0.001 compared with Ethanol group.

We further evaluated the role of PPARα in the development of ethanol induced liver injury by assessing the hepatic expression levels of pro-inflammatory factors phosphatidylinositol 3-kinase (PI3K), OPN and COX-2. We found that ethanol increased the expression of PI3K, OPN and COX-2. Mice treated with WY14643 showed significantly reduced hepatic mRNA and protein expression for PI3K (*P *< 0.001 and *P *< 0.01, respectively), OPN (*P *< 0.001 and *P *< 0.001) and COX-2 (*P *< 0.01 and *P *< 0.05) as compared with mice fed with ethanol liquid diet only (Figure 5). On the other hand, GW6471 further increased PI3K protein (*P *< 0.05) and COX-2 mRNA (*P *< 0.05) expression, but didn't affect OPN expression.

### Activation of PPARα enhanced hepatic expression of adiponectin and heme oxygenase-1 (HO-1)

**Figure 6:**
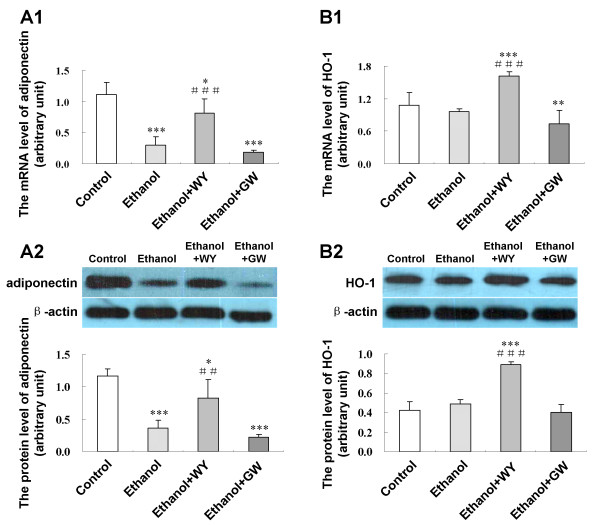
**Effect of PPARα induction by WY14643 on expression of hepatic adiponectin and HO-1**. **(A1) **Expression level of adiponectin mRNA and **(A2) **protein; **(B1) **Expression level of HO-1 mRNA and **(B2) **protein in various treatment groups. Data are expressed as the mean ± SD (n = 6 per group). *P < 0.05, **P < 0.01, ***P < 0.001 compared with Control group; ^##^*P *< 0.01, ^###^*P *< 0.001 compared with Ethanol group.

Hepatic mRNA and protein expression of adiponectin, a major anti-inflammatory adipokine, and HO-1, a key anti-oxidative stress factor, were evaluated by RT-PCR and Western blot. WY14643 up-regulated mRNA and protein expression for adiponectin (*P *< 0.001 and *P *< 0.01, respectively) and for HO-1 (*P *< 0.001 and *P *< 0.001) in mice fed with ethanol liquid diet (Figure 6). GW6471 didn't alter expression of these two genes (Figure 6).

## Discussion

C57BL/6J mice fed with 4% ethanol-containing Lieber-DeCarli liquid diet for 12 weeks showed hepatic steatosis, hepatocyte ballooning, inflammatory cell infiltration and hepatocyte necrosis, accompanied with elevated serum ALT and AST levels. Under transmission electron microscopy, we found that mitochondrial cristae were broken, the membranes were ruptured, and most of the cristae were merged with parts of the membranes. These results indicated that this ALD mouse model mimicked the human alcoholic liver injury.

PPARα, a member of the nuclear hormone receptor superfamily and a receptor for free fatty acid (FFA) [12-14], represses hepatic lipid accumulation by regulating the expression of genes involved in fatty acid synthesis, oxidation and storage. It is also inhibited inflammatory response via inhibiting transcription of nuclear factor-κB and production of inflammatory cytokines [11]. In this study, we found PPARα expression was down-regulated accompanied by the development of ethanol induced hepatic injury. Induction of PPARα by WY14643 treatment for 2 weeks attenuated ethanol induced liver injury, as evidenced by diminished histological steatosis and inflammatory response, improved hepatocyte ultrastructure, as well as decreased serum ALT and AST levels. Administration of PPARα antagonist GW6471 for 2 weeks increased hepatic inflammation. These results indicated that PPARα might play an important protective role in the pathogenesis of ALD.

We further explored the potential mechanisms of the protective effect of PPARα by the use of selective agonist WY14643. Ethanol and its metabolic products impair tricarboxylic acid cycle activity and fatty acid β-oxidation in the mitochondria, resulting in FFA overload and triglyceride accumulation in the liver, ultimately leading to hepatic steatosis. Characteristic PPARα target genes CYP4A10 and CYP4A14 are involved in fatty acid oxidation in a PPARα-dependent manner [15]. Hepatic CYP4A10 and CYP4A14 mRNA expression could be reduced by ethanol and restored by WY14643. FGF21, a hormone primarily produced by liver and fat tissue, was reported to improve lipid metabolism [16-18]. In this study, hepatic FGF21 expression was enhanced by WY14643. In addition, the metabolic effect of FGF21 depends on the activation of its downstream signaling cascade involving the SIRT1 axis [16-18]. The latter had been reported to be responsible for PGC-1α activation and SREBP-1 reduction in the liver [19,20]. We found that WY14643 restored the hepatic expression of SIRT1 which was reduced by alcohol administration in mice. Induction of SIRT1 enhanced mitochondrial oxidative capacity as demonstrated by increases in oxygen consumption and citrate synthase activity, as well as up-regulation of key metabolic genes, including PGC-1α [21], a crucial metabolic mediator for coordinating gluconeogenesis and fatty acid oxidation in the liver [22-24]. In keeping with this observation, induction of PPARα by WY14643 in this study induced the hepatic expression of PGC-1α. Besides, SIRT1 repressed lipogenesis largely by attenuating the activity of SREBP-1 and its downstream gene FAS [20,25-28]. In this connection, downregulation of FAS was demonstrated by WY14643 in this study. The SIRT1 axis has also emerged as a major signaling system in regulating adiponectin signaling in the lipid lowering action [29,30]. We also found that WY14643 increased adiponectin expression and down-regulated hepatic expression of PI3K in ethanol feeding mice, the latter has been reported to participate in the adipogenesis of human mesenchymal stem cells [31] and promote steatosis in cultured hepatocytes exposed to methionine-choline deficient (MCD) medium [32]. Collectively, the anti-steatosis effect of PPARα may be partly related to up-regulation of its target genes CYP4A10 and CYP4A14, induction of SIRT1 axis by up-regulation of FGF21 and adiponectin expression, as well as down-regulation of FAS and PI3K expression, which ameliorated hepatic steatosis induced by ethanol consumption.

Ethanol treatment results in fatty acids accumulation in the liver. Fatty acids are substrate of cytochrome P450 2E1 (CYP 2E1). Induction of CYP 2E1 by fatty acids is a major source of reactive oxygen species (ROS), which evokes oxidative stress and mitochondrial damage in the hepatocytes, in turn worsens fatty acid β-oxidation disorder and hepatic steatosis. Furthermore, ROS activates and sensitizes Kupffer cells, which produce and release pro-inflammatory cytokines, triggering hepatic inflammatory response [11]. PI3K could induce expression of OPN, which increased ALT release by hepatocytes treated with MCD medium [32], indicating that PI3K and OPN were potentially involved in the development of hepatocyte damage. Elevated level of OPN, a chemoattractant molecule, correlated with neutrophil infiltration [4], and neutralizing antibody against OPN prevented neutrophil infiltration and ethanol induced liver injury [5]. Pro-inflammatory factor COX-2 was induced by ethanol as well. In our study, activation of PPARα resulted in the inhibition of these key hepatic inflammatory regulators including PI3K, OPN and COX-2 expression. In this regard, the anti-inflammatory effects of PPARα in ethanol induced steatohepatitis may be related to inhibition of these pro-inflammatory factors.

Besides regulating lipid metabolism in the liver, adiponectin serves as an anti-inflammatory factor. Adiponectin prevents hepatic injury by inhibiting the synthesis and/or release of tumor necrosis factor-α (TNF-α) [33]. HO-1 plays essential role in mediating the anti-inflammatory function of adiponectin. Importantly, after chronic ethanol exposure, induction of HO-1 protects mice from the sensitizing effect of ethanol on LPS-stimulated TNF-α expression [34]. In addition, HO-1 and its down-stream mediator carbon monoxide could increase expression of anti-inflammatory cytokine interleukin-10 in macrophages [35]. In this study, HO-1 expression was up-regulated by PPARα agonist. In keeping with our findings, Otterbein et al. reported that PPARα could regulate HO-1 transcription directly by binding to a PPAR-responsive element in the promoter regions of HO-1 [36]. Thus, PPARα induced HO-1 expression by itself and/or via increasing adiponectin expression may contribute to the amelioration of ethanol induced hepatic inflammatory response.

## Conclusions

The present study showed a protective role of PPARα in ethanol induced liver injury. Activation of PPARα by WY14643 ameliorated hepatic steatosis through increasing lipids oxidation promoting genes CYP4A10, CYP4A14, FGF21, adiponectin, SIRT1 and PGC-1α expression, and suppressing fatty acid synthesis promoting genes FAS and PI3K expression. Induction of PPARα attenuated liver inflammatory response by repressing expression of pro-inflammatory cytokines PI3K, OPN and COX-2, as well as enhancing expression of anti-inflammatory factors adiponectin and HO-1. PPARα agonist administration might serve as an effective therapeutic strategy for ALD.

## Materials and methods

### Animals and treatments

Eight-week-old male C57BL/6J mice with body weight between 20-25 g were obtained from the Experimental Animal Center of Chinese Academy of Medical Sciences, and were bred in a temperature-controlled animal facility with a 12 h light-dark cycle. They had free access to water and were allowed to adapt to their food and environment for 1 week before the start of the experiment. The mice were randomly divided into 4 groups (6 mice per group): 1) Control group: fed with non-alcohol control liquid diet; 2) Ethanol group: fed with 4% ethanol-containing Lieber-DeCarli liquid diet; 3) Ethanol plus WY14643 (Ethanol+WY) group: fed with ethanol liquid diet supplemented with WY14643 (50 mg/kg/d, Cayman Chemical, Ann Arbor, MI, CA); 4) Ethanol plus GW6471 (Ethanol+GW) group: fed with ethanol liquid diet plus GW6471 (10 mg/kg, Tocris bioscience, Bristol, UK), three times/week, intraperitoneal injection. The ethanol concentration was raised gradually from 2% to 4% within 2 weeks, and then the mice were fed a 4% ethanol-containing Lieber-DeCarli liquid diet for 12 weeks. PPARα agonist WY14643 and antagonist GW6471 were administrated in the last 2 weeks. Control mice were fed with the same volume of a control liquid diet prepared by replacing ethanol in the Lieber-DeCarli liquid diet with isocaloric maltose. Animals were sacrificed after overnight fasting at the end of experiment. Blood samples were collected from femoral artery for biochemical analysis. Livers were weighed, frozen or fixed in 10% formalin for histological analysis, fixed in 4% glutaraldehyde for electron microscopy, or snap-frozen in lipid nitrogen followed by storage at -80°C in a freezer until required. All the protocols and procedures were performed following the guidelines of the Hebei Committee for Care and Use of Laboratory Animals and were approved by the Animal Experimentation Ethics Committee of the Hebei Medical University.

### Biochemical analysis

Serum ALT and AST levels were measured by enzymatic method using an automatic biochemical analyzer (Olympus UA2700, Japan) according to the manufacturer's instructions.

### Histological analysis

Haematoxylin and eosin stained paraffin-embedded liver sections (5 μm thick) were scored as follows: (a) degree of steatosis (0 ≤ 10%, 1 = 10-33%, 2 = 33-66%, 3 ≥ 66%); (b) degree of hepatocyte ballooning (0 = none, 1 = mild and moderate, 2 = severe); (c) degree of necroinflammation (0 = none, 1 = mild, 2 = moderate, 3 = severe) in accordance with a scoring system for ALD designed by Dominguez et al. [37].

### Transmission electron microscopy (TEM) examination

The tissue fixed in 4% glutaraldehyde solution was postfixed in 1.5% osmium tetroxide solution, dehydrated, and fixed firmly into polybed resin. Micro-thin sections were performed and stained with lead citrate and uranyl acetate for a Hitachi model 7500 TEM examination.

### Quantitative real-time reverse transcription polymerase chain reaction (qRT-PCR) analysis of hepatic mRNA expression

Total RNA was isolated from liver tissues using Trizol Reagent (Tiangen Biotech, Beijing, China) according to the manufacturer's instructions. The hepatic mRNA levels of PPARα, CYP4A10, CYP4A14, FGF21, SIRT1, PGC-1α, FAS, PI3K, OPN, COX-2, adiponectin and HO-1 were determined by qRT-PCR using the ABI PRISM 7500 sequence detection system (Applied Biosystems, Foster, CA) with SYBR Green Reagent (Tiangen Biotech). Expression levels of the target genes were normalized against an endogenous reference gene glyceraldehydes 3-phosphate dehydrogenase (GAPDH). The specific primer sequences were listed in Table 1. All data were obtained using Sequence Detector Software (Applied Biosystems).

**Table 1:** Primer sequences used for real-time RT-PCR analysis

Gene	Product length	Primer sequences
PPARα	149 bp	F 5'-GATGTCACACAATGCAATTCG-3'
		R 5'-GGTAGGCTTCGTGGATTCTCT-3'
CYP4A10	64 bp	F 5'-ACACTGCTCCGCTTCGAACT-3'
		F 5'-CAAGTCGGGCTAAGGGCA-3'
CYP4A14	81 bp	F 5'-AAGGCAGTGTTCAGTTGGATG-3'
		F 5'-GGCGAAAGAAAGTCAGGTTGT-3'
FGF21	199 bp	F 5'-CAGATGACGACCAAGACACTG-3'
		R 5'-TCAAAGTGAGGCGATCCATAG-3'
SIRT1	197 bp	F 5'-ACGCTGTGGCAGATTGTTATTA-3'
		R 5'-GCAAGGCGAGGCATAGATACC-3'
PGC-1α	109 bp	F 5'-AGGAACAGCAGCAGAGACAAAT-3'
		R 5'-CTGGGGTCAGAGGAAGAGATAA-3'
FAS	193 bp	F 5'-GGGTTCTAGCCAGCAGAGTCTA-3'
		R 5'- TGAGGTTGCTGTCGTCTGTAGT-3'
PI3K	198 bp	F 5'- GCACGGCGATTACACTCTTAC-3'
		R 5'-TTGGACACTGGGTAGAGCAAC-3'
OPN	121 bp	F 5'-GGTGATAGCTTGGCTTATGGAC-3'
		R 5'-CCTTAGACTCACCGCTCTTCAT-3'
COX-2	122 bp	F 5'-GCCTTCTCCAACCTCTCCTACT-3'
		R 5'-ACCTTTTCCAGCACTTCTTTTG-3'
adiponectin	202 bp	F 5'-CCAGTATCAGGAAAAGAATGTGG-3'
		R 5'-TGGTGTATGGGCTATGGGTAGT-3'
HO-1	427 bp	F 5'-AACAAGCAGAACCCAGTCTATG-3'
		R 5'-TGAGCAGGAAGGCGTCTTA-3'
GAPDH	233 bp	F 5'-GGTGAAGGTCGGTGTGAACG-3'
		R 5'-CTCGCTCCTGGAAGATGGTG-3'

Abbreviations: PPARα, peroxisome proliferator activated receptor alpha; CYP4A10, cytochrome P450 4A10; CYP4A14, cytochrome P450 4A14; FGF21, fibroblast growth factor 21; SIRT1, Sirtuin 1; PGC-1α, PPAR gamma coactivator 1α; FAS, fatty acid synthase; PI3K, phosphatidylinositol 3-kinase; OPN, osteopontin; COX-2, cyclooxygenase 2; HO-1, heme oxygenase-1; GAPDH, glyceraldehyde-3- phosphate dehydrogenase.

### Western blot analysis of hepatic protein expression

Total protein was extracted and concentration was measured by the Bradford method (DC protein assay; Bio-Rad, Hercules, CA). Equal amounts of protein (100 μg/well) were loaded onto 12% SDS-PAGE for each sample and proteins were transferred onto equilibrated polyvinylidene difluoride membranes (Millipore Corporation, Billerica, MA) by electroblotting. The membranes were incubated with primary antibodies of PPARα, FGF21, SIRT1, PGC-1α, FAS, PI3K, OPN, COX-2, adiponectin, HO-1, and β-actin (Santa Cruz Biotechnology, Santa Cruz, CA), respectively, overnight at 4°C. Membranes were further incubated with secondary antibody for 1 h at room temperature. Proteins were detected by enhanced chemiluminescence (Santa Cruz Biotechnology). The amount of protein expression was corrected by that of β-actin in the same sample and the bands were quantified by scanning densitometry using the digital Kodak Gel Logic 200 (Carestream Molecular Imaging, Woodbridge, CT).

### Statistical analysis

All data are expressed as mean ± standard deviation (SD). Statistical analysis on the data was performed by one-way analysis of variance (ANOVA) or Kruskal-Wallis *H *test, with the least significant difference-*t *(LSD-*t*) test or mann-whitney *u *test for post-hoc comparison using SPSS 13.0 (v. 13.0; SPSS Inc., Chicago, IL), and *P-*value below 0.05 was considered significant.

## List of Abbreviations

ALD: alcoholic liver disease; ALT: alanine aminotransferase; AST: aspartic transaminase; COX-2: cyclooxygenase-2; CYP4A10: cytochrome P450 4A10; CYP4A14: cytochrome P450 4A14; FAS: fatty acid synthase; FFA: free fatty acid; FGF21: fibroblast growth factor 21; HO-1: heme oxygenase-1; OPN: osteopontin; PGC-1α: proliferators activated receptor gamma coactivator 1 alpha; PI3K: phosphatidylinositol 3-kinase; PPARα: Peroxisome proliferator activated receptor alpha; SIRT1: Sirtuin 1; SREBP-1: sterol regulatory element binding protein 1.

## Competing interests

The authors declare that they have no competing interests. The authors alone are responsible for the content and writing of the paper.

## Authors' contributions

YN designed the research; LK, WR, WL, SZ, HM, RW, YZ and WW performed the experiments; LK and YN analyzed data; YN, LK and JY wrote the paper. All authors read and approved the final manuscript.
